# Albuminuria or proteinuria in glomerular disease and CKD—which one to use?

**DOI:** 10.1093/ndt/gfag020

**Published:** 2026-02-17

**Authors:** Vanja Ivković, Andreas Kronbichler, Chee Kay Cheung, Jonathan Barratt, Annette Bruchfeld, Jürgen Floege, Augusto Vaglio

**Affiliations:** Department of Health, Medicine and Caring Sciences, Linköping University, Linköping, Sweden; Department of Nephrology, Hypertension, Dialysis and Transplantation, University Hospital Center Zagreb, Zagreb, Croatia; Faculty of Health Studies, University of Rijeka, Rijeka, Croatia; Department of Health, Medicine and Caring Sciences, Linköping University, Linköping, Sweden; Department of Internal Medicine IV, Nephrology and Hypertension, Medical University Innsbruck, Innsbruck, Austria; Mayer IgA Nephropathy Laboratories, Department of Cardiovascular Sciences, University of Leicester, Leicester, UK; John Walls Renal Unit, University Hospitals Leicester NHS Trust, Leicester, UK; Mayer IgA Nephropathy Laboratories, Department of Cardiovascular Sciences, University of Leicester, Leicester, UK; John Walls Renal Unit, University Hospitals Leicester NHS Trust, Leicester, UK; Department of Health, Medicine and Caring Sciences, Linköping University, Linköping, Sweden; Department of Renal Medicine, Karolinska University Hospital and CLINTEC Karolinska Institutet, Stockholm, Sweden; Department of Nephrology and Department of Cardiology, RWTH Aachen University Hospital, Aachen, Germany; Department of Biomedical, Experimental and Clinical Sciences “Mario Serio”, University of Florence, Florence, Italy; Nephrology and Dialysis Unit, Meyer Children’s Hospital IRCCS, Florence, Italy

## ALBUMINURIA AND PROTEINURIA IN CHRONIC KIDNEY DISEASE

**Figure 1:**
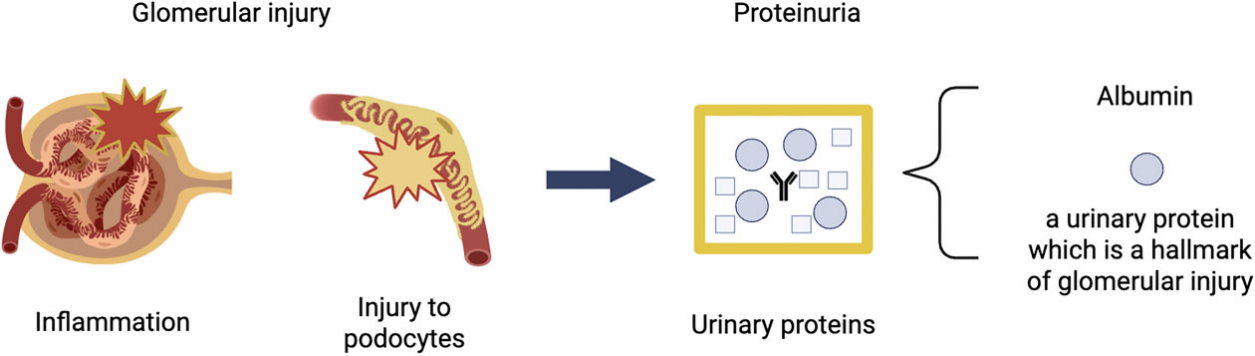
Glomerular injury, such as due to inflammation, immune-mediated processes, podocytopathy or other causes of nephron loss, leads to albuminuria which is a hallmark of glomerular disease.

The presence of protein and albumin in the urine is a hallmark of kidney disease, with albuminuria in particular being a marker of glomerular injury [1]. While small amounts of albumin are filtered in the healthy kidney, almost all of it is reabsorbed, mostly in the proximal tubule and specifically in its first section [2, 3]. Albuminuria develops through a multistep process involving damage to the podocytes and/or glomerular basement membrane and the loss of endothelial barrier selective permeability due to inflammation or other damage [1] (Fig. 1). Evidence from experimental studies suggests that albumin may be toxic to tubules. It has been shown to have proinflammatory effects, lead to reactive oxygen species accumulation and oxidative damage, and lysosome- and endoplasmic reticulum stress–mediated damage, and promote fibrosis [4].

Proteinuria and albuminuria are most frequently measured using a semiquantitative dipstick test (which has limited reliability) or spot urines normalized to urinary creatinine [5]. The gold standard is an attempted 24-h urine collection with determination of UPCR or UACR [5, 6]. The reliability of albuminuria dipsticks is limited and might only be useful for an initial assessment [7]. Determination of the urinary albumin-to-creatinine ratio (UACR) is more costly than the urinary protein-to-creatinine ratio (UPCR), but data supporting the 2014  National Institute for Health and Care Excellence (NICE) chronic kidney disease (CKD) guideline suggests that it is more cost-effective when compared with UPCR and that even slight increases in the diagnostic accuracy of UACR compared with UPCR justify the higher cost [5, 8–10]. Moreover, standardization for urine albumin measurement is more easily achievable than for total urine protein measurement due to the complexity of non-albumin protein content [11].

**Figure 2:**
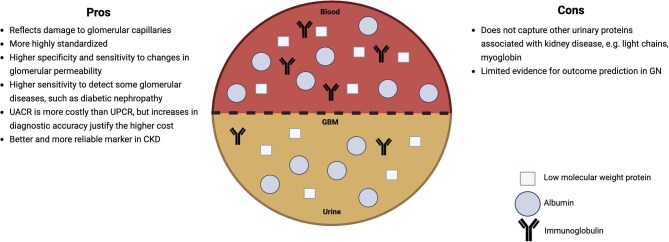
Pros and cons of albuminuria in diagnosis and prognosis of kidney diseases. The figure shows a schematic of a glomerulus and the filtration of low molecular proteins, albumin and high molecular weight immunoglobulins in glomerular disease. Also shown are the pros and cons of the measurement of albuminuria compared with proteinuria.

In contrast, total proteinuria consists of albumin and other higher and lower molecular weight proteins, such as immunoglobulins, light chains and β2-microglobulin. It is more widely used in clinical practice and research, and may reflect processes other than glomerular injury [12]. Large studies showed that albuminuria is a better and more reliable marker in CKD [13–17]. Based on that evidence, the 2012 KDIGO clinical practice guidelines for the evaluation and management of CKD recommended a shift from proteinuria to albuminuria as one of the two main criteria, in addition to estimated glomerular filtration rate (eGFR), to classify and define CKD stages [18, 19]. Albuminuria is a more logical and plausible endpoint from a physiological and pathophysiological standpoint, however the recommendation was also based on evidence showing that measurement of urinary albumin is more standardized, specific and sensitive, thus it has been carried over to the newest edition of the guidelines published in 2024 [17–19] (Fig. 2).

## ALBUMINURIA AS A SURROGATE ENDPOINT IN GLOMERULAR DISEASES

Almost a decade and a half later, that shift is yet to happen in glomerular diseases as proteinuria remains the key indicator used in clinical work and research. Diabetes-related kidney disease, which is not primarily immune-mediated, is a long-standing exception as long-term practices linked to treatment of diabetes have always favored the measurement of albuminuria [20]. In contrast, proteinuria remains the main benchmark in trials across glomerular diseases, where it dictates both patient inclusion and outcome measures [21]. A search of ClinicalTrials.gov for randomized controlled trials (RCT) in glomerular diseases having albuminuria as a primary, secondary or other endpoint showed that albuminuria was an endpoint in only 65 out of 901 (7.2%) trials, 49 of which were conducted in the last 10 years. In contrast, 281 (31.2%) trials had proteinuria as an outcome.

Interestingly, two recent meta-analyses which provide the strongest evidence to date for albuminuria as surrogate endpoint in CKD also support its use in glomerular diseases. A meta-analysis of individual-patient data from 48 RCTs in CKD which enrolled 85 861 patients (<2% had glomerular disease) evaluated whether albuminuria is a valid surrogate endpoint for kidney failure showed that each 30% reduction in the geometric mean of the UACR in the trial treatment group relative to the control group was associated with a 19% lower hazard for the clinical endpoint (kidney failure or doubling of serum creatinine). There was no clear evidence that the association of UACR and outcome could vary by CKD etiology [diabetes and unspecified cause vs immunoglobulin A nephropathy (IgAN), being the only included glomerulonephritis, as there were only three trials included in other glomerular diseases] [22]. Accepting the small numbers, the association was stronger in IgAN compared with other causes of CKD, suggesting that UACR may be a more optimal surrogate endpoint. While IgAN patients contributed only 1% of the patients in the meta-analysis, still 1037 patients from seven IgAN trials were included. The other meta-analysis which included 148 994 patients from 38 research and clinical cohorts compared UACR and UPCR in predicting kidney failure and reported that the association was stronger with UACR. Moreover, this association was even more pronounced in patients with glomerular diseases, 3% of the total patients included (*N* = 4681 with a total of 689 kidney failure events), as well as in those with higher UACR and more severe kidney disease [23].

Three studies published recently in *NDT* and *CKJ* add important observations to the association between albuminuria and outcomes in IgAN. These studies try to find answers to similar questions, but a deeper insight reveals that there are remarkable differences in the profiles of included patients (Table 1) [24–26]. A study by Faucon *et al*. [24] enrolling 1269 IgAN patients from Sweden evaluated an older, mostly male population with advanced CKD and higher albuminuria levels. By contrast, Yu *et al*. [25] report on 1839 IgAN patients from China with a relatively lower baseline risk profile (Table 1). Despite apparent differences in risk profiles, both studies confirm prior findings from the UK National Registry of Rare Kidney Diseases (RaDaR) and other studies reporting that IgAN has a dire long-term prognosis [27–29]. Most importantly, both found that UACR was strongly and incrementally associated with kidney outcomes. Even patients with UACR from 0.3 to 0.5 g/g had a >1.5-fold higher risk of >30% or higher decline in eGFR or kidney failure compared with those with UACR <0.3 g/g (namely, 1.6-fold in the Swedish study and 2.9-fold in the Chinese study). Patients with a UACR ≥1.5 g/g had a 4- to 6-fold higher risk for at least a 30% eGFR decline or kidney failure. Furthermore, in patients with serial UACR measurements, ACR trajectories were strongly associated with differences in outcomes, confirming the degree of albuminuria as a modifiable risk factor for disease progression [30]. No comparisons were made between the predictive value of albuminuria compared with proteinuria. This is in line with previous studies which showed that proteinuria >0.5 g/day portends a worse prognosis [27–29]. They also confirm the findings of an earlier, 2024 German CKD cohort study by Stamellou *et al*. [26] that enrolled 421 patients with a risk profile somewhere between the two mentioned studies, in which 120 (28.5%) patients reached a composite endpoint of >40% eGFR decline or kidney failure after 6.5 years follow-up (Table 1). ACR was the strongest predictor with hazard ratios, depending on ACR category, from 2.03 to 5.64, compared with the lowest stratum with ACR <0.1 g/g [26]. The data from these three studies provide key evidence for albuminuria as a risk factor for kidney failure over a continuum of values, including those with very low albuminuria, and a valid and crucial outcome across heterogeneous populations and risk strata, but it is not possible to compare whether there is an additive value of measuring albuminuria compared with proteinuria.

While studies directly comparing albuminuria and proteinuria in glomerular diseases are rare, there is limited evidence that UACR may outperform both UPCR and 24-h proteinuria in predicting eGFR decline or kidney failure in IgAN and might improve the predictive value when added to the model containing traditional risk factors [31, 32]. ACR might also be more useful in a chronic setting, as one small single-center study from China reported it might be a better choice than 24-h proteinuria to follow up patients with IgAN. In this study, ACR was associated with anemia, acidosis, hypoalbuminemia, hyperphosphatemia, hyperkalemia, hypercholesterolemia and higher cystatin C, while 24-h proteinuria was only associated with hypoalbuminemia and hypercholesterolemia. ACR was also associated with the extent of interstitial fibrosis and tubular atrophy, key indicators of chronicity [33].

Albuminuria also predicted outcomes in antineutrophil cytoplasmic antibody–associated vasculitis (AAV) as patients with UACR >300 mg/g at 6 months after induction treatment had poor kidney function recovery compared with those with UACR 300 mg or lower with an adjusted mean difference in 5-year ΔeGFR of –12.5 mL/min/1.73 m^2^. Patients with albuminuria after induction were more often in the Berden mixed or crescentic class than focal or sclerotic classes and had a 6.5-fold higher adjusted hazard of kidney failure [34]. These findings highlight the importance of the timing of albuminuria measurement and suggest that albuminuria at 6 months might mostly be an indicator of long-term glomerular damage and inflammation which contributes to the development of fibrosis and, consequently, CKD. ADVOCATE (A Phase 3 Clinical Trial of CCX168 (Avacopan) in Patients With ANCA-Associated Vasculitis), a trial of the efficacy and safety of avacopan in AAV, is one of few exceptions, having albuminuria and not proteinuria as an endpoint [35]. Trial findings showed that avacopan was efficient in reducing UACR and that early reduction of albuminuria saves kidney function, at least in the short-term.

While no discernible difference in the predictive value of UACR versus UPCR was evident in lupus nephritis (LN), it is plausible that the measurement of albuminuria might lead to earlier diagnosis and may show a stronger association with cardiovascular outcomes [36]. Of note, even LN patients with little or no proteinuria (<0.5 g/24 h) had some degree of albuminuria [37]. Patients with high activity (defined as class III, IV or V) had higher urinary albumin than those with class I or II and albuminuria was independently associated with proliferative and class V LN [37].

**Table 1:** Comparison of three recently published studies on albuminuria in IgA nephropathy.

	Study/Country		
Characteristics	Faucon *et al.* (2025) Sweden	Stamellou *et al.* (2024) Germany	Yu *et al.* (2025) China
Patients (N), sex (%)	1269, 74.1% men	421, 67.0% men	1839, 47.5% men
Age (years)	53	52	38
eGFR (mL/min/1.73 m^2^)	33	53	84
CKD stage (%)	CKD 4: 45.3 CKD 5: 11.4	CKD 4: 9.8 CKD 5: 0.2	CKD 4: 2.8 CKD 5: 0
ACR (g/g)	0.7	0.4	0.48
ACR>1.5 g/g (%)	25.1	13^a^	15.9
Hematuria	Not reported	Not reported	Not reported
Oxford classification (MEST-C score)			
*M1*	Not reported	Not reported	10.0%
*E1*			5.3%
*S1*			71.5%
*T1-T2*			11.2%
*C1-C2*			Not reported
Follow-up time (years)	5.5 years	6.5 years	2.2 years (?)
Primary outcome (%)	↓eGFR_30_ 41.6% KRT 40.7%^b^	↓eGFR_40_ 15.2% ESKD 13.3%^d^	↓eGFR_30_ or ESKD 11.8%^c^
eGFR change (mL/min/1.73 m^2^ per year)	–3.06	Provided only for ACR categories	−1.90
eGFR change by ACR category (mL/min/1.73 m^2^ per year)			
≥2 g/g	−2.41	−3.16^e^	−2.41
1.5-2 g/g	−2.60		−2.60
1.0-1.5 g/g	−2.14	−2.34^e^	−2.14
0.5-1.0 g/g	−1.83		−1.83
0.3-0.5 g/g	−1.93	−1.49^e^	−1.93
<0.3 g/g	−1.37	−0.90^e^	−1.37
Patients with serial ACR measurements (%)	61.9	Not available	23.5
HR for MAKE by ACR category			
≥2 g/g	4.5	5.0^f^	5.6
1.5-2 g/g	4.2	5.6^f^	6.2
1.0-1.5 g/g	2.8	3.8^f^	3.1
0.5-1.0 g/g	2.0		2.4
0.3-0.5 g/g	1.6	2.0^f^	2.9
<0.3 g/g	Reference	Reference^f^	Reference

a ≥ 1.4 g/g. Primary outcome.

b composite of eGFR decline of over 30% (↓eGFR_30_) or kidney replacement therapy.

c composite of eGFR decline of over 30% (↓eGFR_30_) or ESKD (defined as eGFR < 15 mL/min/1.73 m^2^ or kidney replacement therapy.

d composite of eGFR decline of over 40% ((↓eGFR_40_), eGFR <15 ml/min/1.73 m^2^ and initiation of kidney replacement therapy.

e Reported as: ≥ 1.4 g/g, 0.6–1.4 g/g, 0.1–0.6 g/g and <0.1 g/g.

f Reported as: >2.2 g/g, 1.4-2.2 g/g, 0.6-1.4 g/g, 0.1-0.6 g/g and <0.1 g/g. Abbreviations: ACR=albumin-to-creatinine ratio; ↓eGFR_30_=decrease in eGFR over 30%; ↓eGFR_40_=decrease in eGFR over 40%; ESKD=end-stage kidney disease; HR=hazard ratio; KRT=kidney replacement therapy; MAKE=major adverse kidney event.

## CONCEPT OF CHRONIC KIDNEY DISEASE IN GLOMERULAR DISEASES

Results of a recent study in which CKD was defined as an eGFR <60 mL/min/1.73 m^2^ or albuminuria ≥30 mg/24 h in at least two consecutive tests, spaced ≥3 months apart, highlight the importance of considering CKD in glomerular disease. Among 175 patients with systemic lupus erythematosus (SLE), 101 (57.8%) had CKD. Surprisingly, 52.8% of non-dialysis-dependent SLE patients had only albuminuria and no reduced eGFR. Furthermore, 46.1% of SLE patients with CKD had no history of LN [38]. While the study has several limitations, mainly due to single-center, retrospective design, the findings are in line with data from the Swedish Renal Registry which showed that 83% of LN patients have eGFR <60 mL/min/1.73 m^2^ and 84.6% of patients had a UACR >30 mg/g (category A2 or A3) [39]. In contrast, studies which only considered decreased eGFR significantly underestimated the prevalence of CKD in SLE with rates frequently around 20% or lower [40]. While albuminuria might be a better marker of kidney involvement in SLE, diagnosis and management still remain centered on proteinuria, with an important distinction of new-onset or worsening proteinuria—which may mark flares—versus persistent proteinuria, which is frequently a reflection of damage accrual [41, 42]. Despite this, albuminuria is not discussed in any major LN guidelines, including the 2025 EULAR update or the 2025 ACR guidelines which are both clearly focused on proteinuria as an outcome [43–45].

The importance of CKD as a consequence of glomerular disease was disregarded in the 2019 EULAR/ERA-EDTA guidelines, but has been incorporated in the latest 2025 EULAR update [43, 46]. This recent update includes a recommendation dedicated to CKD informing the risk of progression and the need for interdisciplinary management, which includes monitoring of and treating risk factors for CKD. Another recommendation clearly states that “prevention of CKD progression is crucial to avoid the long-term sequelae of kidney failure” [43]. In line with this, the 2025 KDIGO IgAN guidelines address the need for a dual approach in IgAN patients at risk for kidney function loss which includes tackling both IgAN-specific drivers for nephron loss (stopping synthesis of pathogenic forms of IgA and formation of immune complexes, as well as kidney injury mediated by them) and generic responses to nephron loss (reduction of glomerular hyperfiltration proteinuria and the impact of proteinuria on the tubulointerstitium and blood pressure control) [47]. These recommendations are essential and warranted across the spectrum of glomerular diseases, given the very high proportion of glomerular disease patients having various stages of CKD and reaching kidney failure [27, 48–50]. Disease modification is crucial in kidney disease and the ERA Immunonephrology Working Group has recently proposed a definition of disease-modifying anti-nephropathic drug (DMANDs) which are used in the management of immune-mediated glomerulonephritis and podocytopathies to minimize disease activity, prevent loss of kidney structure and function, and reduce treatment-related toxicity [51].

There are, however, several points which should be a part of future research agendas regarding albuminuria in glomerular disease. Due to a lack of large studies directly comparing albuminuria with proteinuria, definitive evidence for whether UACR adds value above measuring UPCR in guiding treatment decisions or, indeed, in predicting prognosis across different glomerular diseases is absent [5]. How UACR performs in patients with heavy proteinuria and/or nephrotic syndrome should be evaluated further as well as the effect of non-selective and non-albuminuric proteinuria, which portend a worse prognosis in some glomerular diseases [52–54]. The use of both UPCR and UACR may be appropriate in some settings, such as in patients with advanced kidney disease or specific diseases, for example those which increase non-albumin proteinuria. Future studies should explore whether the association between UACR and kidney failure in glomerular diseases is comparable in patients receiving disease-modifying or antiproteinuric therapy beyond predominantly hemodynamically acting drugs such as renin–angiotensin–aldosterone system inhibitors, sodium-glucose transport protein inhibitors, endothelin receptor antagonists and mineralocorticoid receptor antagonists. Finally, precise biomarkers that, when combined with proteinuria/albuminuria can help distinguish active disease from other drivers of kidney function deterioration, are much warranted [55].

The place of albuminuria measurement in the management of glomerular diseases is receiving increasing attention. The use of albuminuria in glomerular disease management deserves further evaluation in light of evidence from meta-analyses of recent trials, albeit the majority of included trials were in non-glomerular diseases, and the increasing acceptance that glomerular diseases are an important cause of CKD [56, 57]. However, the path from proteinuria to albuminuria as a key guide of treatment decisions in glomerulonephritis must be evidence based, with properly conducted comparative studies in individual glomerular diseases, and will likely be a slow and gradual one. Current clinical trial activity in the glomerular disease space is greater than ever before, and the hope is that the data generated from these studies will allow comparison between albuminuria and proteinuria in predicting treatment response and short-term prognosis in well-defined patient cohorts. These data, may in the future, inform the drug regulators and nephrologists on the value of measuring albuminuria in the management of glomerular diseases.
